# Necrotizing Fasciitis of the Labia Caused by Beta-Hemolytic Streptococcus and Streptococcus Anginosus: A Case Report and Literature Review

**DOI:** 10.7759/cureus.93038

**Published:** 2025-09-23

**Authors:** Zachary Bowens, Hannah Flynn, Jordan Winebrenner, Michaeleena Carr, Lindsay Tjiattas-Saleski

**Affiliations:** 1 Simulation, Edward Via College of Osteopathic Medicine Carolinas, Spartanburg, USA; 2 Medicine, Edward Via College of Osteopathic Medicine Carolinas, Spartanburg, USA; 3 Emergency Medicine, PRISMA Health Tuomey Hospital, Sumpter, USA; 4 Emergency Medicine, Edward Via College of Osteopathic Medicine Carolinas, Spartanburg, USA

**Keywords:** beta hemolytic streptococcus, case report, fournier's gangrene, necrotizing fasciitis, soft tissue infection, streptococcus anginosus

## Abstract

Necrotizing fasciitis (NF) presents a formidable clinical challenge due to its rapid progression and potentially fatal outcomes. This case report describes the presentation, management, and outcomes of a 33-year-old female with advanced perineal NF. The report underscores the importance of prompt recognition, surgical debridement, and targeted antimicrobial therapy in optimizing patient outcomes. An extensive literature review was conducted to provide additional context, focusing on the epidemiology, pathophysiology, diagnostic modalities, and treatment strategies for NF. Notably, this case highlights a novel synergistic relationship between beta-hemolytic *Streptococcus *and *Streptococcus anginosus*, contributing to the evolving understanding of NF pathogenesis. Collaborative interdisciplinary care remains paramount for improving survival in this complex and life-threatening condition.

## Introduction

This work was presented at the American College of Osteopathic Obstetricians and Gynecologists (ACOOG) as a poster presentation (04/26-31/2023) and at the Edward Via College of Osteopathic Medicine (VCOM) - Carolinas Campus Research Recognition Symposium (on September 2, 2024).

Necrotizing fasciitis (NF) is a rare but serious soft tissue infection that can progress rapidly if not promptly diagnosed and treated with broad-spectrum antibiotics and surgical debridement. Since the early 2000s, the mortality rate due to NF has been reduced by approximately 50%. Since this reduction, the mortality rate has remained at 20% and has not significantly changed [1]. NF has an incidence that ranges between 0.18 and 1.55 per 100,000 persons, and a population-level mortality rate that ranges between 0 and 0.3 per 100,000 persons per year. Among those who develop NF, worse outcomes have been associated with advanced age, prolonged hospital stays, and female sex, although the disease affects men and women equally overall [2,3]. NF infects and erodes the interstitial and perimuscular fascia and subcutaneous adipose tissue. It has a predilection for spreading along muscular fascia due to its poor blood supply; however, it does not tend to infect the underlying muscular tissue due to its rich blood supply. This differentiates NF from necrotizing myositis (NM), which is an infection of skeletal muscle generally caused by group A Streptococcus (GAS). NF of the genital, perineal, or perianal regions is referred to as Fournier’s gangrene (FG). It is classified as either monomicrobial (type 2), which is most commonly caused by GAS and occurs in those with no underlying comorbidities, or polymicrobial (type 1), which usually occurs in older individuals with comorbidities [3,4].

Predisposing factors

NF can occur in those with and without predisposing risk factors [4]. While there are no definitive risk factors for NF, some associated factors include diabetes mellitus, obesity, pelvic surgical procedures, hemorrhoids, immunosuppression, blunt trauma, muscle injuries, and burns [5-7]. Patients with a history of diabetes mellitus have been observed to have a more severe infectious course than those without. This has been particularly evident in NF cases of the distal extremities and perineum [4]. NF is more prevalent in cases of uncontrolled diabetes mellitus with poor genital hygiene and comorbidities and less prevalent in cases of controlled diabetes mellitus without other risk factors. Polymicrobial cases of NF often occur in the hospital setting; therefore, risk factors for hospitalization, such as those mentioned previously, predispose individuals to increased risk of NF [8]. Additionally, pharmacologic treatments of diabetes, such as sodium-glucose cotransporter 2 inhibitors (SGLT2i), have been implicated in severe cases of NF. While a causal link between NF and SGLT2i has not been established, it is believed that SGLT2i, in addition to the other risk factors discussed here, may contribute to the development of NF. This is due to the increase in observed incidences of NF in patients with diabetes mellitus, with comorbidities and other risk factors, who also began SGLT2i therapy shortly before (approximately nine months) developing NF. Furthermore, delayed diagnosis of NF can occur particularly in those taking nonsteroidal anti-inflammatory drugs (NSAIDs), as these can mask the manifestations of inflammation, thereby delaying diagnosis and treatment.

Common causes

NF type 2 (monomicrobial) is commonly caused due to infection by gram-positive cocci such as *Staphylococcus *and *Streptococci *strains and is generally caused by minor penetrating traumas and breaches in the skin. NF type 1 (polymicrobial) is typically due to infections by a combination of gram-negative and gram-positive aerobic and anaerobic bacteria. It is associated with immunocompromised states, diabetes, and mucosal breaches, often associated with surgical interventions [3]. The pathologic agents often associated with NF are associated with numerous pathologies; however, one notable association is vaginitis.

Vaginitis is characterized by a deficiency in the amount of *Lactobacillus *species in the genital mucosa where it serves a protective function by contributing to the acidic environment of the vagina. Outside of sexually transmitted infections, vaginitis is commonly caused by group B *Streptococcus*, *Enterococcus faecalis*, *Escherichia coli*, *Staphylococcus aureus*, *Gardnerella vaginalis*, and *Atopobium vaginae*; however, *Streptococcus anginosus *has recently been implicated as a common cause [9].

Group B *Streptococcus *is a gram-positive aerobic and facultative anaerobic streptococcus that grows in long and short chains on blood agar with Todd-Hewitt broth. While group B *Streptococcus *infection is typically associated with pregnancy, it has been seen to cause systemic symptoms in the elderly, diabetic, and immunocompromised individuals. Similarly, nontraumatic causes of *S. anginosus* infections are associated with diabetes mellitus, alcoholism, drug abuse, and hematological or gastrointestinal malignancies. Many of the common causes of vaginitis are also causes of NF. Thus, in predisposed populations, there is potential for recurrent bacterial vaginosis and aerobic vaginitis to seed NF; however, there is insufficient evidence to establish a causal link between them.

NF can present alone with symptoms of sepsis, characterized by fever, tachycardia, altered mental status, and diabetic ketoacidosis, and/or with cutaneous manifestations, such as cellulitis. While NF is a clinical diagnosis, it can be definitively diagnosed via histological analysis of tissue obtained during surgical exploration [8]. The decision to undergo surgery can be aided by radiography and laboratory studies.

Radiographic modalities such as radiographic films and computed tomography can be used to identify gross swelling, inflammation, and subcutaneous gases located within tissues and are particularly valuable when a retained foreign body or other occult source is suspected. However, imaging is not required to proceed to surgery when clinical suspicion is high, and waiting for radiographic confirmation can delay the timely debridement essential for survival. Currently, no validated radiographic criteria exist to grade disease severity or to determine operative versus nonoperative management. Laboratory tests such as complete blood counts (CBC), complete metabolic panels (CMP), coagulation studies, creatinine kinase (CK) levels, and C-reactive protein (CRP) levels may be used to identify leukocytosis with neutrophilia, acidosis, altered renal function, and inflammatory markers. These abnormalities are commonly associated with advanced cases of NF [6]. Additionally, tools such as the Laboratory Risk Indicator for Necrotizing Fasciitis (LRINEC) may be used to obtain a quantitative estimate of risk. While the validity and reliability of the LRINEC score have been debated, it may aid in providing clarity when used together with the entire clinical picture [10-12].

Treatment and prognosis

Hemodynamic support, antimicrobial therapy, and surgical exploration and debridement are essential first steps in the treatment of NF [8,13,14]. Surgical exploration is necessary and remains the primary therapeutic modality for the definitive diagnosis and initial treatment of NF [8]. In the absence of surgical debridement, mortality approaches 100% [6,15]. Most cases of NF are type 2 (polymicrobial) and should be empirically treated with broad-spectrum antibiotics that cover aerobic, methicillin-resistant *Staphylococcus aureus* (MRSA), and anaerobic species and may consist of clindamycin and an aminoglycoside or fluoroquinolone [8,13-16].

After biopsy and culture, targeted antimicrobial treatment should be given [3,5-8,15,16]. The two salient complications associated with NF are necrotizing mediastinitis and vascular pathologies (thrombosis, hemorrhage, and necrosis). NM has been principally associated with increased mortality; however, immunocompromised states and diabetes mellitus have not been seen to increase the risk of NM [5]. The largest contributor to increased morbidity and mortality is delayed diagnosis and treatment. There are currently no guidelines for the early diagnosis of NF. Therefore, the diagnosis is dependent upon manifestations of infection that occur late in the disease course and a high index of suspicion. Patients contribute to the delay in diagnosis and treatment by failing to recognize the severity of NF in its early stages. Delay in diagnosis, and therefore treatment, may be due to the similarity of the early stages of NF with cellulitis and other cutaneous infections. At present, the recommendations for early recognition, diagnosis, and treatment of NF are for healthcare professionals to maintain a high index of suspicion and develop an understanding of the various manifestations of NF [15]. This demonstrates the need for objective measures and guidelines for the early diagnosis of NF.

## Case presentation

The patient was a 33-year-old African American female who presented to the emergency department with generalized body aches and a right-sided labial abscess that had worsened over the past few days. She denied vomiting and upper respiratory infection symptoms. Upon presentation, she was fatigued, but well developed, well nourished, alert, and oriented times three with a blood pressure of 180/92 (left arm sitting), pulse of 100 bpm, temperature of 98°F/36.7°C, respiratory rate of 19 bpm, weight of 118 kg (260 lbs), SpO_2_ of 100%, and a BMI of 39.53 kg/m².

**Figure 1:**
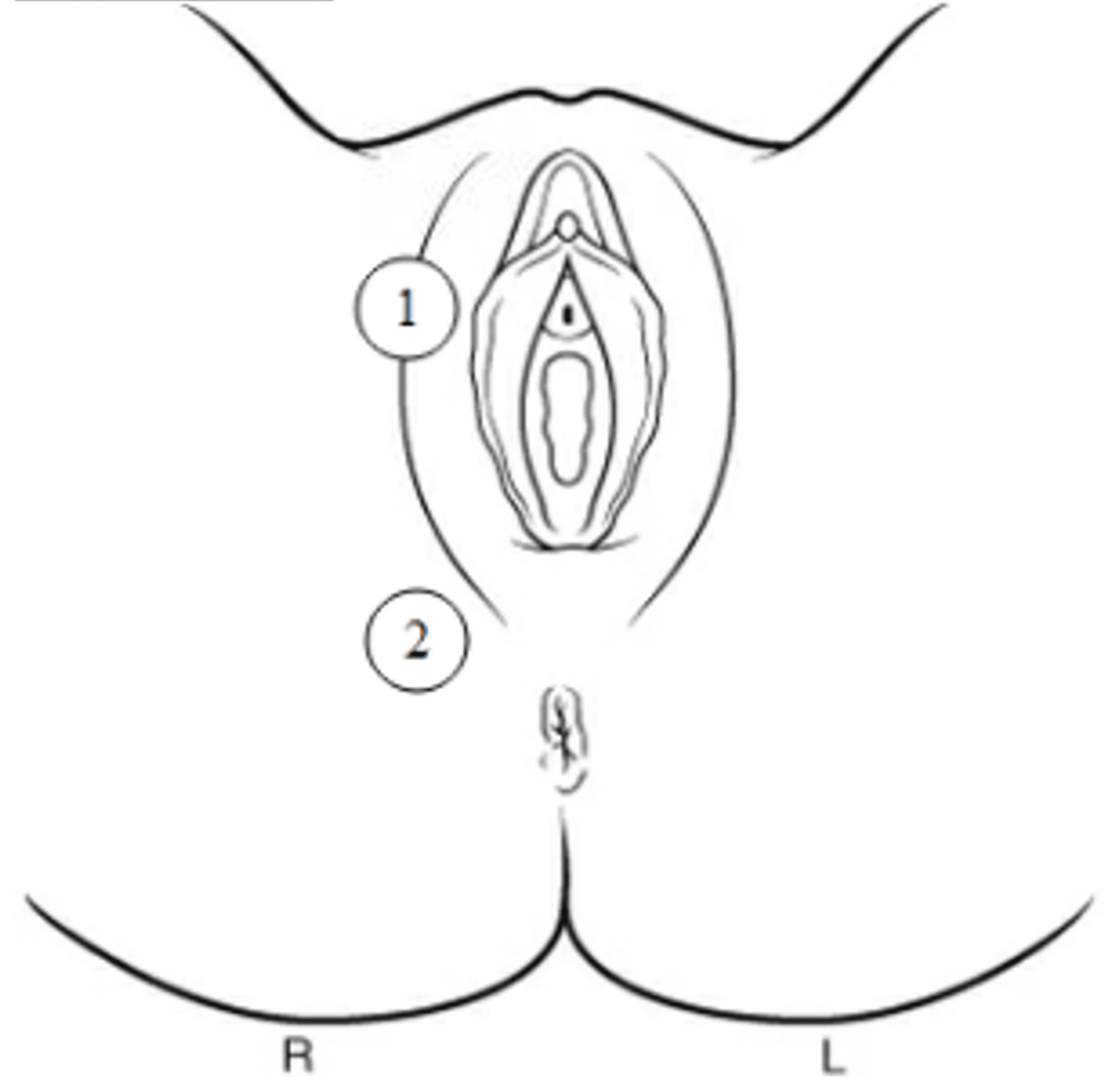
Anatomical locations of induration and erythema in the vulvar and perineal regions An anatomical diagram illustrating areas of induration and erythema observed in our patient. (1) Right labia majora. (2) Perineal region. Both sites demonstrated significant induration and erythema. Image credit: This figure was created by the authors.

The patient’s past medical history was notable for morbid obesity, hypertension, diabetes mellitus, diabetic foot ulcers, and iron deficiency anemia. Medications included metformin, lisinopril, and famotidine. The patient’s physical exam was normal, except that she was obese, and her right labia and right posterior lateral perineum were indurated and erythematous. The right labia was also tender to palpation, and there was no opening or drainage (Figure 1).

**Figure 2:**
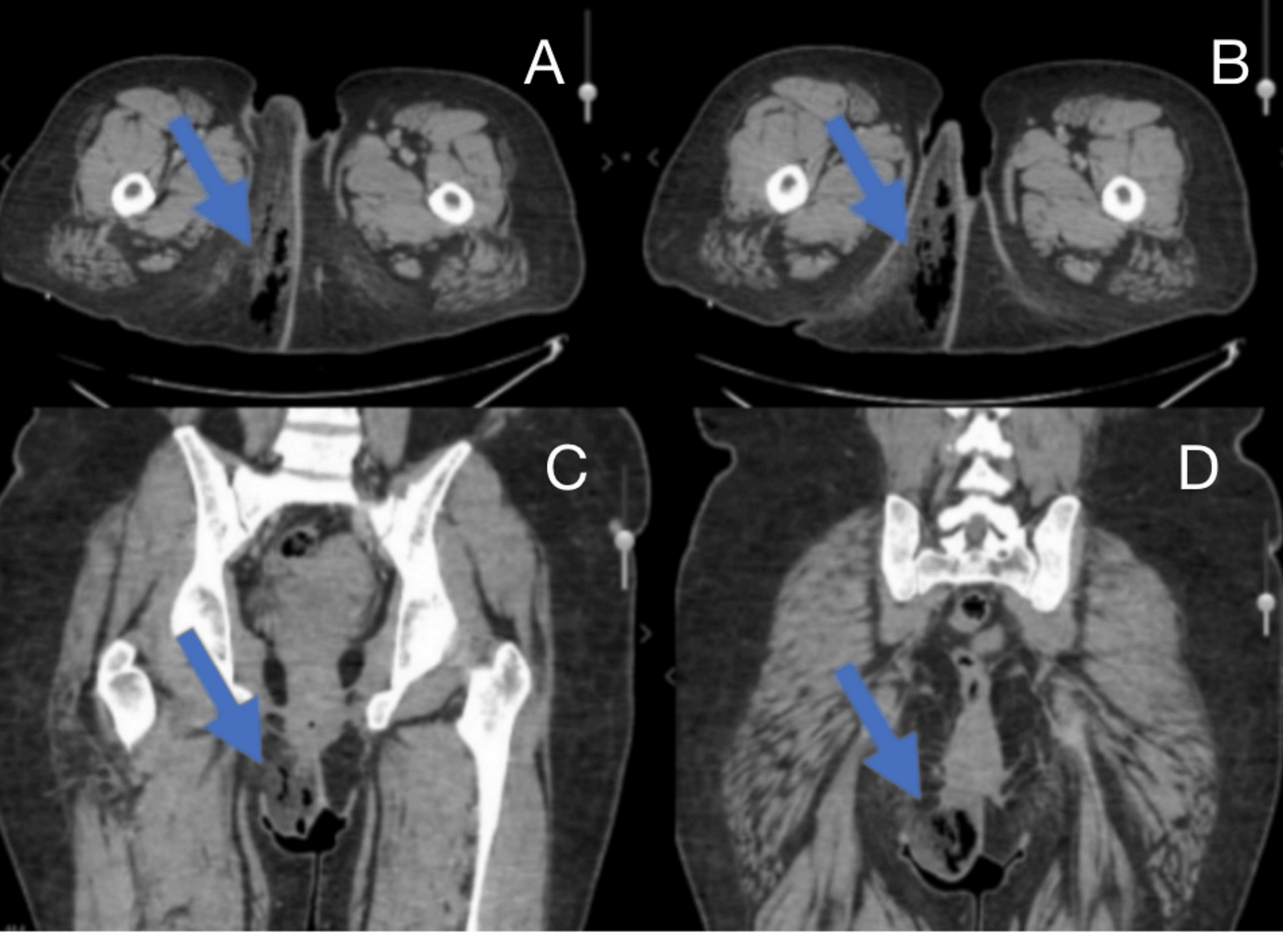
Patient's computed tomography scan Computed tomography (CT) images demonstrating soft tissue changes in the perineal and vulvar regions. (A) Transverse CT scan, cephalad level, showing right labial induration and subcutaneous fat stranding (arrow). (B) Transverse CT scan, more caudal section, showing progression of inflammation into the perineal region (arrow). (C) Coronal CT scan demonstrating involvement of the right labia majora and perineum (arrow). (D) Coronal CT scan showing inferior extension of inflammatory changes toward the posterior perineum (arrow).

The emergency department workup included a blood culture, pregnancy test (HCG Scr), urine analysis (UA), venous lactic acid, urine culture, urine microscopy, CBC with differential, urinalysis with reflex microscopy, basic metabolic panel (BMP), COVID/FLU/respiratory syncytial virus (RSV) testing, and a computed tomography (CT) scan without contrast (Figure 2). The laboratory results revealed a urinary tract infection, with a urine culture >100,000 colony-forming units (CFU), WBC (UA) 5-10, and bacteria (UA) 3+. Upon further analysis, the patient’s urine had trace leukocytes and was positive for nitrites, blood, and protein (100). Additionally, urine glucose (≥2000) and ketones (40) were elevated. The patient’s BMP showed low sodium - 134 (136-144 mmol/L), low CO_2_ - 19 (23-29 mmol/L), and elevated glucose - 329 (64-100 mg/dL). The patient’s CBC with differential revealed microcytic anemia with a mean corpuscular volume (MCV) of 74.1% (79.0-92.2 fL), WBC of 16 (4.3-9.1 10³/mm³), RBC of 3.48 (4.63-6.08 10⁶/mm³), hemoglobin of 7.6 (13.7-17.5 g/dL), and hematocrit of 25.8 (40.1%-51.0%).

The CT scan revealed no evidence of bowel obstruction, no signs of lymphadenopathy, normal abdominal aorta caliber, no free fluid, normal urinary bladder and pelvic reproductive organs, and no osseous abnormalities. The soft tissue of the body wall was significant for thickening, fat stranding, and edema with gas within the soft tissue of the right labia and glute. There was no drainable fluid observed. Intravenous fluids were administered (30 cc/kg per sepsis protocol) as well as antibiotics, including clindamycin (900 mg), vancomycin load dose, and piperacillin and tazobactam (3.375 g).

Upon admission to surgical services, the patient’s status remained stable. The patient was taken to surgery for excision and debridement of a necrotizing soft tissue infection with gas gangrene of the right labia and buttocks. An incision was made in the indurated and erythematous right labia and buttock area. Fluid was collected for cultures. With the extension of the incision, a considerable amount of gas was released from the wound. The incision extension included the full length of necrotic tissue (18 cm). The entire area of necrotic tissue (18 x 7 x 5 cm) was excised and discarded. Hemostasis was achieved, the wound was packed with Curlex gauze dampened with Dakin’s solution, and a dry dressing was placed over the wound. The patient was extubated and taken to recovery in stable condition. The fluid taken from the wound during surgery was cultured. Notably, the aerobic culture was positive for beta-hemolytic *Streptococcus *(GBS) and *S. anginosus*.

Plan and analysis

The patient presented to the emergency department with an advanced case of vulvar/perianal NF. Together, the patient’s physical exam and laboratory results were highly suggestive of NF and sufficient for diagnosis. The radiological study, while not necessary for diagnosis and treatment, supports the clinical suspicion of NF.

The administered antibiotics should empirically include those with a broad spectrum, such as beta-lactams. As such, the administration of piperacillin and tazobactam was appropriate. Multidrug therapy may consist of high-dose penicillin, clindamycin, aminoglycosides, and vancomycin. The patient was given clindamycin, vancomycin, piperacillin, and tazobactam before tissue culture. In addition to the prescribed antibiotics, administering an aminoglycoside would have been appropriate for gram-negative coverage; however, clindamycin along with piperacillin and tazobactam provided robust gram-negative coverage. Subsequent to antibiotic administration, surgical wound debridement and culture biopsy were appropriate. Adjustment of antimicrobial therapy based on tissue culture was not indicated in this report; however, this would be a logical next step in addition to standard wound care.

Differential diagnosis

The differential diagnosis for NF includes arterial thrombosis, clostridial myonecrosis, iliofemoral phlebothrombosis, NM, Bartholin gland cyst, obliterating atherosclerosis, pyomyositis, superficial vulvar abscess, vulvar cellulitis, and vulvar erysipelas.

Support for conditions and additional investigations

During the early stages of a necrotizing soft tissue infection in the vulva, the diagnosis can usually be made based on the clinical presentation alone. Additional investigations, such as ultrasound, CT scan, or radiography, are only needed when there is uncertainty about the clinical picture, and no significant signs of systemic inflammation are observed. Tissue biopsy is not a reliable method for diagnosing necrotizing infections. It is crucial to consider a wide range of potential conditions, both infectious and noninfectious, that may present with similar clinical features (acute cellulitis, severe pain, high leukocytosis or leukopenia, tachycardia, and shock) when establishing a differential diagnosis for necrotizing soft tissue infection. If suspicions of a necrotizing infection persist, it is recommended to surgically examine all soft tissue layers [2].

Additionally, using a prognostic tool such as the LRINEC (Table 1) [17] may prevent marked morbidity; however, there is debate about its ability to predict mortality in cases of necrotizing fasciitis [10-12].

**Table 1:** Laboratory risk indicator for necrotizing fasciitis (LRINEC) with the patient's values Score ≤ 5 = <50% risk (low); Score 6-7 = intermediate risk (medium); Score ≤ 8 = >75% risk (high). Total LRINEC score of the patient: 6 (intermediate risk).

Test	Patient’s Value	LRINEC Value	LRINEC Score
Hb (g/dl)	7.6	>13.5	0
		11–13.5	1
		<11	2
WBC (10³/µL)	16	<15	0
		15–25	1
		>25	2
Sodium (mmol/L)	134	≥135	0
		<135	2
Creatinine (µmol/L)	0.93	≤1.6	0
		>1.6	2
Glucose	329	≤180	0
		>180	1
C-reactive protein (mg/L)	N/A	≤150	0
		>150	4

Pathophysiology

NF occurs when bacteria enter the underlying soft tissue through fissures in the skin layer [18]. Bacteria will spread rapidly along muscle fascia due to its reduced blood supply, destroying vascular and neural structures [18]. Eventually, tissue ischemia and necrosis will occur, resulting in sepsis and death [18].

Tissue destruction will be caused by a wide range of virulence factors solely determined by the pathogens responsible for the infection. Bacteria responsible for NF type 2 (monomicrobial) include group A *Streptococci *(GAS) species and *Staphylococcus *species, which produce an exotoxin to serve as their virulence factor [19,20]. Microbes in NF type 1 (polymicrobial) are a combination of aerobic and anaerobic bacteria [18]. The *Clostridium *species have been implicated as a common cause in type 1 NF and utilize an alpha toxin to cause necrosis [20].

Regardless of the type of NF a patient has, when virulence factors encounter tissues, cytokines are released [19]. Cytokines are largely responsible for cellular recruitment and increasing inflammation within the microvasculature [19]. This recruitment will allow microthrombi to form, leading to worsened ischemia and necrosis of the affected tissues, further propagating the spread of the pathogen [19].

Patient management and treatment

Imaging

Imaging can be a useful adjunct to aid in the diagnosis of NF; however, it should not be used as the sole diagnostic measure [19]. GAS can be seen on plain film radiological studies for patients too unstable to travel for advanced studies; however, plain film x-ray serves no purpose in diagnosing NF [18,19]. CT should be considered as the initial imaging modality used in stable patients when the clinical diagnosis is unclear [19]. CT imaging of NF will show edema along the fascia plane, although edema may not be visualized in earlier stages of the disease process [20]. Imaging should not be utilized if it will delay surgical intervention [18].

Surgical Management

Early identification of NF is imperative to positive patient outcomes [18]. In cases where a diagnosis of NF is suspected, surgical intervention should not be delayed [18,19]. A definitive diagnosis can only be obtained with surgical exploration [19]. All necrotic tissue will need aggressive surgical debridement with wide margins, and in some patients, multiple surgeries may be needed to achieve this [18,19]. Surgical judgment is crucial when removing tissue that may not appear necrotic [18]. If normal-appearing tissue is ever in question, the tissue should be removed [18]. Early surgical management remains the most important intervention for patient outcomes [19]. Delay will only increase mortality and increase the number of operations patients need to control the infection [19].

Medical Management

Patients with suspected NF should be started on aggressive broad-spectrum empirical antibiotics while undergoing surgical assessment [19]. An empirical antibiotic regimen should have coverage for polymicrobial infections and include agents to cover gram-positive, gram-negative bacteria, and MRSA [19]. Tissue samples and blood cultures should be taken for gram stain, culture, and sensitivity testing to tailor the antibiotic treatment [20]. The patient should remain on empirical antibiotics until the pathogen responsible for the infection has been identified and a new antibiotic regimen can be started [20].

## Discussion

NF is commonly associated with immunosuppression, diabetes mellitus, and traumatic injuries; however, diabetes mellitus has been put forward as one of the most common predisposing factors [7]. NF type 2 (monomicrobial) is commonly caused by *Staphylococcus *and *Streptococci *infections associated with dermal breaches. NF type 1 (polymicrobial) is commonly caused by a combination of gram-negative and gram-positive aerobic and anaerobic infections associated with superficial mucosal breaches, diabetes mellitus, and immunocompromised states [3]. It is suggested that the rapid course of NF type 1 is due to synergism between the causative agents [9]. This patient’s culture biopsy was positive for beta-hemolytic *Streptococcus *(GBS) and *S. anginosus*. *S. anginosus *has been shown to exhibit synergistic activity with *Edwardsiella tarda*; however, it has not been seen with GBS [9].

*S. anginosus *virulence gene sag is chiefly responsible for lysis of vaginal epithelial cells, contributing to the disruption of the vaginal mucosa. This provides a possible mechanism of synergistic activity.

The diagnosis of NF is primarily clinical; however, early diagnosis is difficult due to the wide variety of early cutaneous findings, which may mimic those of cellulitis or abscesses. Even when broad-spectrum antibiotics are administered, the diagnosis of NF usually occurs after progression of the disease process [4]. This supports the importance of early wound biopsy, culture, and surgical debridement. Without these early interventions, mortality rates approach 100% [6]. This delay in diagnosis allows for the development of significant sequelae that can be exacerbated by comorbidities such as diabetes mellitus and immunocompromised states. Therefore, a delay in the diagnosis of NF contributes to the morbidity and mortality of the disease course [21]. There is no indication that the patient presented in this work sought medical care in the early stages of the disease process. It may be that the patient did not recognize the severe nature of her condition early in the disease process. This, combined with its rapid course, may explain the advanced presentation.

NF has an incidence that ranges between 0.18 and 1.55 per 100,000 persons, and a mortality rate that ranges between 0 and 0.3 per 100,000 persons [2,3]. It has also been observed that its incidence tends to increase with increasing age. Mortality is higher among patients with advanced age, diabetes, immunocompromised states, or delays in surgical debridement. Incidence rises with age as well, with a marked increase in middle-aged adults and a slightly higher occurrence in males [2].

The most significant complications of NF are associated with markedly increased mortality rates. In those with streptococcal infections, toxic shock syndrome (38% mortality rate) and septic shock (45% mortality rate) represent two of the most serious complications. This patient presented with septicemia; however, it had not progressed to septic shock, as indicated by her vital signs. The prompt treatment of this patient with fluids, appropriate antimicrobial therapy, and surgical debridement indicates a favorable prognosis. Complications may arise from poor wound healing due to her diagnosis of diabetes mellitus. Therefore, careful consideration should be given to wound management, which may require a prolonged hospital stay.

This case report is subject to certain limitations. Due to the uncommon and unique characteristics of this particular case, it is challenging to extrapolate the report’s findings broadly. Therefore, this case underscores the significance of conducting a thorough evaluation of a patient’s medical history, physical examination, and imaging studies to ensure precise diagnoses, especially when faced with highly varied presentations.

## Conclusions

This case contributes to the literature on NF of the perineal region in adult patients, demonstrates the importance of prompt intervention and workup of NF in patients with comorbidities, and presents a new synergistic relationship between beta-hemolytic *Streptococcus *(GBS) and *S. anginosus *in NF type 1. The patient presented with septicemia in the advanced stages of the disease process. Hemodynamic stabilization, empiric pharmacological treatment, surgical debridement, and wound culture were vital steps in obstructing the disease process and preventing further exacerbation via the patient’s comorbidities.
